# 4,6-Dichloro-2-((*E*)-{4-[(*E*)-3,5-dichloro-2-hy­droxy­benzyl­idene­amino]­butyl­imino}­meth­yl)phenol

**DOI:** 10.1107/S1600536812028693

**Published:** 2012-06-30

**Authors:** Hadi Kargar, Reza Kia, Amir Adabi Ardakani, Muhammad Nawaz Tahir

**Affiliations:** a Department of Chemistry, Payame Noor University, PO Box 19395-3697 Tehran, I. R. of IRAN; b Department of Chemistry, Science and Research Branch, Islamic Azad University, Tehran, Iran; c Ardakan Branch, Islamic Azad University, Ardakan, Iran; d Department of Physics, University of Sargodha, Punjab, Pakistan

## Abstract

The asymmetric unit of the title compound, C_18_H_16_Cl_4_N_2_O_2_, comprises half of a potentially tetra­dentate Schiff base ligand. It is located about a twofold rotation axis that bis­ects the central C—C bond of the butane-1,4-diamine group. There are two intra­molecular O—H⋯N hydrogen bonds making *S*(6) ring motifs. In the crystal, mol­ecules are linked by pairs of weak C—H⋯Cl inter­actions, forming inversion dimers, which are further connected by C—H⋯O hydrogen bonds into two-dimensional frameworks that lie parallel to (001).

## Related literature

For standard bond lengths, see: Allen *et al.* (1987 ▶). For hydrogen-bond motifs, see: Bernstein *et al.* (1995 ▶). For related Schiff base ligands, see: Kargar *et al.* (2011 ▶); Kia *et al.* (2010 ▶).
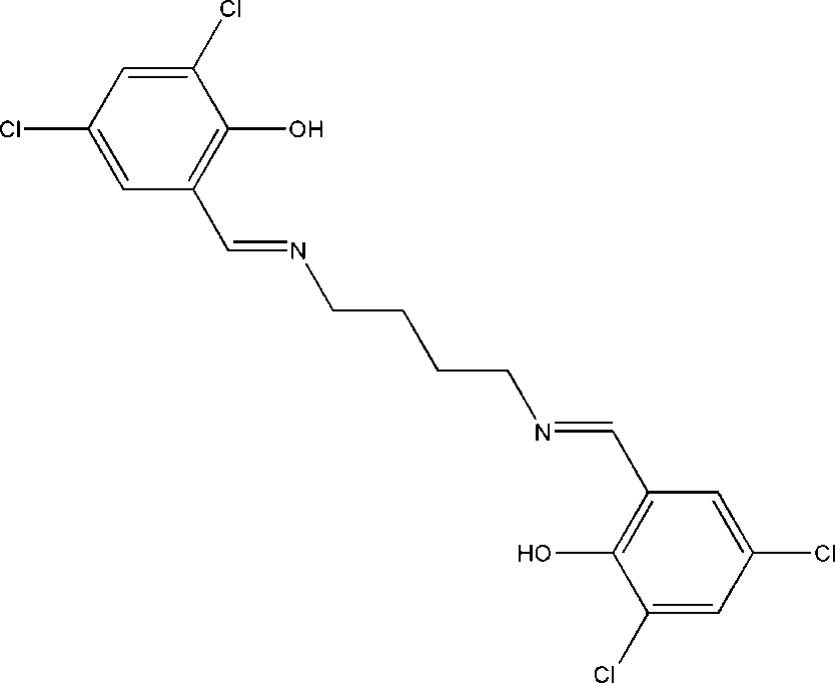


## Experimental

### 

#### Crystal data

C_18_H_16_Cl_4_N_2_O_2_
*M* *_r_* = 434.13
Orthorhombic, 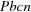

*a* = 15.871 (3) Å
*b* = 12.505 (3) Å
*c* = 9.4133 (18) Å
*V* = 1868.3 (6) Å^3^
*Z* = 4
Mo *K*α radiation
μ = 0.65 mm^−1^
*T* = 291 K
0.35 × 0.16 × 0.14 mm

#### Data collection

Bruker SMART APEXII CCD area-detector diffractometer
Absorption correction: multi-scan (*SADABS*; Bruker, 2005 ▶) *T*_min_ = 0.805, *T*_max_ = 0.915
8772 measured reflections
2057 independent reflections
1264 reflections with *I* > 2σ(*I*)
*R*_int_ = 0.058

#### Refinement

*R*[*F* ^2^ > 2σ(*F* ^2^)] = 0.045
*wR*(*F* ^2^) = 0.095
*S* = 1.02
2057 reflections
118 parameters
H-atom parameters constrained
Δρ_max_ = 0.31 e Å^−3^
Δρ_min_ = −0.27 e Å^−3^

### 

Data collection: *APEX2* (Bruker, 2005 ▶); cell refinement: *SAINT* (Bruker, 2005 ▶)’; data reduction: *SAINT*; program(s) used to solve structure: *SHELXS97* (Sheldrick, 2008 ▶); program(s) used to refine structure: *SHELXL97* (Sheldrick, 2008 ▶); molecular graphics: *SHELXTL* (Sheldrick, 2008 ▶); software used to prepare material for publication: *SHELXTL* and *PLATON* (Spek, 2009 ▶).

## Supplementary Material

Crystal structure: contains datablock(s) global, I. DOI: 10.1107/S1600536812028693/su2460sup1.cif

Structure factors: contains datablock(s) I. DOI: 10.1107/S1600536812028693/su2460Isup2.hkl

Supplementary material file. DOI: 10.1107/S1600536812028693/su2460Isup3.cml

Additional supplementary materials:  crystallographic information; 3D view; checkCIF report

## Figures and Tables

**Table 1:** Hydrogen-bond geometry (Å, °)

*D*—H⋯*A*	*D*—H	H⋯*A*	*D*⋯*A*	*D*—H⋯*A*
O1—H1⋯N1	0.95	1.71	2.565 (3)	149
C5—H5⋯O1^i^	0.93	2.48	3.370 (3)	160
C3—H3⋯Cl2^ii^	0.93	2.83	3.738 (3)	165

Symmetry codes: (i) 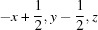; (ii) 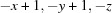.

**Fig. 1.**
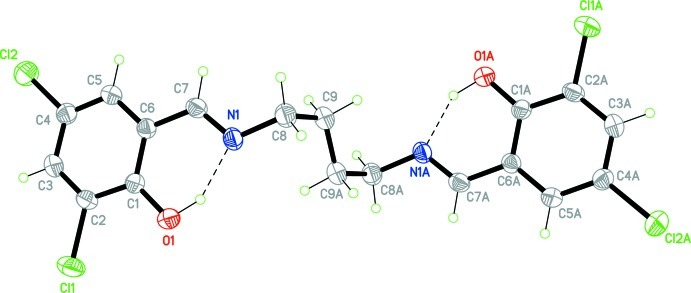
The molecular structure of the title compound, showing 40% probability displacement ellipsoids and the atomic numbering [symmetry code for suffix A = -x, -y + 1, -z + 1].

**Fig. 2.**
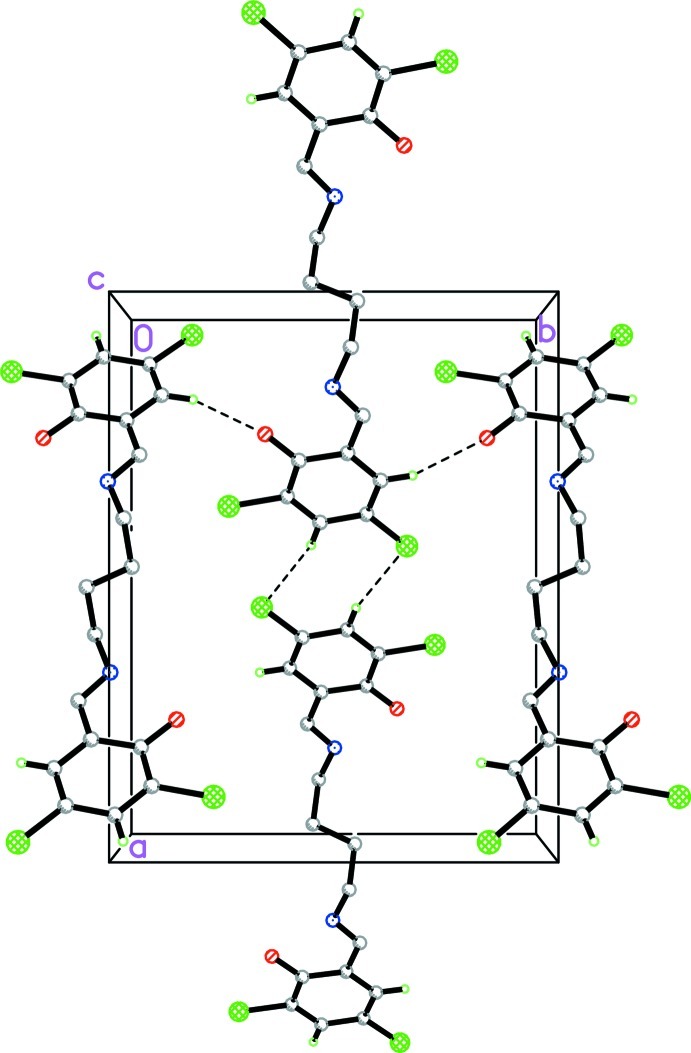
The crystal packing of the title compound showing the C—H···O and weak C—H···Cl interactions (dashed lines; see Table 1 for details) forming two dimensional networks. Only the H atoms involved in hydrogen bonding are shown.

## supplementary crystallographic information

### Comment

In continuation of our work on the crystal structure analyses of Schiff base ligands (Kargar *et al.*, (2011); Kia *et al.*, (2010), we report herein on the crystal structure of the title compound. The asymmetric unit of the title compound, Fig. 1, comprises half of a potentially tetradentate Schiff base ligand. It is located about a two-fold rotation axis that bisects the central C9-C9a bond of the butane-1,4-diamine group. The bond lengths (Allen *et al.*, 1987) and angles are within the normal ranges. The intramolecular O—H···N hydrogen bonds make *S(6)* ring motifs (Fig. 2 and Table 1; Bernstein *et al.*, 1995).

In the crystal, molecules are linked by pairs of weak C—H···Cl interactions to form inversion dimers which are further connected by C—H···O hydrogen bonds along the *b* axis direction, forming two dimensional networks that lie parallel to the ab plane (Table 1 and Fig. 2).

### Experimental

The title compound was synthesized by adding 3,5-dichlorosalicylaldehyde (2 mmol) to a solution of butylenediamine (1 mmol) in ethanol (30 ml). The mixture was refluxed with stirring for 30 min. The resultant solution was filtered. Yellow-needle single crystals of the title compound, suitable for *X*-ray structure determination, were obtained by recrystallization from ethanol by slow evaporation of the solvents at room temperature over several days.

### Refinement

The OH H atom was located in a difference Fourier map and was constrained to ride on the parent O atom with U_iso_(H) = 1.5U_eq_(O). The C-bound H-atoms were included in calculated positions and treated as riding atoms: C—H = 0.93 and 0.97 Å for CH and CH_2_ H-atoms, respectively, with U_iso_(H) = 1.2U_eq_(C).

### Crystal data

**Table:**

C_18_H_16_Cl_4_N_2_O_2_	*F*(000) = 888
*M**_r_* = 434.13	*D*_x_ = 1.543 Mg m^−^^3^
Orthorhombic, *P**b**c**n*	Mo *K*α radiation, λ = 0.71073 Å
Hall symbol: -P 2n 2ab	Cell parameters from 851 reflections
*a* = 15.871 (3) Å	θ = 2.8–27.5°
*b* = 12.505 (3) Å	µ = 0.65 mm^−^^1^
*c* = 9.4133 (18) Å	*T* = 291 K
*V* = 1868.3 (6) Å^3^	Needle, yellow
*Z* = 4	0.35 × 0.16 × 0.14 mm

### Data collection

**Table:**

Bruker SMART APEXII CCD area-detector diffractometer	2057 independent reflections
Radiation source: fine-focus sealed tube	1264 reflections with *I* > 2σ(*I*)
Graphite monochromator	*R*_int_ = 0.058
φ and ω scans	θ_max_ = 27.1°, θ_min_ = 2.1°
Absorption correction: multi-scan (*SADABS*; Bruker, 2005)	*h* = −20→12
*T*_min_ = 0.805, *T*_max_ = 0.915	*k* = −16→15
8772 measured reflections	*l* = −10→12

### Refinement

**Table:**

Refinement on *F*^2^	Primary atom site location: structure-invariant direct methods
Least-squares matrix: full	Secondary atom site location: difference Fourier map
*R*[*F*^2^ > 2σ(*F*^2^)] = 0.045	Hydrogen site location: inferred from neighbouring sites
*wR*(*F*^2^) = 0.095	H-atom parameters constrained
*S* = 1.02	*w* = 1/[σ^2^(*F*_o_^2^) + (0.0259*P*)^2^ + 0.896*P*] where *P* = (*F*_o_^2^ + 2*F*_c_^2^)/3
2057 reflections	(Δ/σ)_max_ < 0.001
118 parameters	Δρ_max_ = 0.31 e Å^−^^3^
0 restraints	Δρ_min_ = −0.27 e Å^−^^3^

### Special details

**Table:**

Geometry. All esds (except the esd in the dihedral angle between two l.s. planes) are estimated using the full covariance matrix. The cell esds are taken into account individually in the estimation of esds in distances, angles and torsion angles; correlations between esds in cell parameters are only used when they are defined by crystal symmetry. An approximate (isotropic) treatment of cell esds is used for estimating esds involving l.s. planes.
Refinement. Refinement of F^2^ against ALL reflections. The weighted R-factor wR and goodness of fit S are based on F^2^, conventional R-factors R are based on F, with F set to zero for negative F^2^. The threshold expression of F^2^ > 2sigma(F^2^) is used only for calculating R-factors(gt) etc. and is not relevant to the choice of reflections for refinement. R-factors based on F^2^ are statistically about twice as large as those based on F, and R- factors based on ALL data will be even larger.

### Fractional atomic coordinates and isotropic or equivalent isotropic displacement parameters (Å^2^)

**Table:**

	*x*	*y*	*z*	*U*_iso_*/*U*_eq_	
Cl1	0.37204 (6)	0.74164 (6)	0.18745 (9)	0.0673 (3)	
Cl2	0.44335 (5)	0.32968 (6)	0.08251 (9)	0.0613 (3)	
O1	0.24660 (12)	0.65449 (13)	0.3845 (2)	0.0492 (5)	
H1	0.2062	0.6189	0.4413	0.074*	
N1	0.16734 (13)	0.50222 (17)	0.5089 (2)	0.0383 (5)	
C1	0.29214 (16)	0.57913 (19)	0.3202 (3)	0.0356 (6)	
C2	0.35462 (17)	0.60699 (19)	0.2224 (3)	0.0404 (7)	
C3	0.40082 (17)	0.5318 (2)	0.1515 (3)	0.0428 (7)	
H3	0.4418	0.5526	0.0866	0.051*	
C4	0.38606 (17)	0.4244 (2)	0.1772 (3)	0.0413 (7)	
C5	0.32680 (17)	0.3932 (2)	0.2738 (3)	0.0402 (7)	
H5	0.3181	0.3209	0.2910	0.048*	
C6	0.27940 (16)	0.46897 (19)	0.3465 (3)	0.0344 (6)	
C7	0.21558 (16)	0.4352 (2)	0.4470 (3)	0.0379 (6)	
H7	0.2096	0.3628	0.4670	0.046*	
C8	0.10183 (16)	0.4624 (2)	0.6055 (3)	0.0435 (7)	
H8A	0.0946	0.5125	0.6833	0.052*	
H8B	0.1194	0.3944	0.6452	0.052*	
C9	0.01787 (17)	0.4483 (2)	0.5280 (3)	0.0481 (8)	
H9A	0.0258	0.3993	0.4492	0.058*	
H9B	−0.0225	0.4163	0.5926	0.058*	

### Atomic displacement parameters (Å^2^)

**Table:**

	*U*^11^	*U*^22^	*U*^33^	*U*^12^	*U*^13^	*U*^23^
Cl1	0.0782 (6)	0.0361 (4)	0.0874 (6)	−0.0057 (4)	0.0253 (5)	0.0114 (4)
Cl2	0.0628 (5)	0.0484 (4)	0.0727 (6)	0.0047 (4)	0.0223 (4)	−0.0103 (4)
O1	0.0497 (12)	0.0349 (10)	0.0630 (12)	0.0051 (9)	0.0161 (11)	0.0044 (9)
N1	0.0308 (12)	0.0419 (12)	0.0421 (13)	−0.0032 (11)	0.0016 (11)	0.0050 (11)
C1	0.0305 (15)	0.0343 (13)	0.0420 (15)	0.0009 (12)	−0.0014 (13)	0.0019 (12)
C2	0.0410 (17)	0.0321 (13)	0.0481 (17)	−0.0052 (13)	0.0009 (14)	0.0058 (12)
C3	0.0362 (16)	0.0458 (16)	0.0465 (17)	−0.0041 (13)	0.0077 (14)	0.0027 (13)
C4	0.0394 (17)	0.0371 (14)	0.0475 (16)	−0.0003 (13)	0.0042 (14)	−0.0056 (13)
C5	0.0408 (16)	0.0324 (13)	0.0473 (17)	−0.0022 (12)	0.0036 (14)	0.0013 (12)
C6	0.0309 (15)	0.0353 (13)	0.0371 (15)	−0.0014 (12)	−0.0007 (12)	0.0026 (11)
C7	0.0354 (16)	0.0330 (13)	0.0453 (16)	−0.0042 (13)	−0.0035 (13)	0.0041 (12)
C8	0.0383 (16)	0.0475 (16)	0.0446 (16)	−0.0011 (14)	0.0032 (15)	0.0056 (13)
C9	0.0408 (18)	0.0456 (16)	0.0578 (19)	−0.0038 (14)	0.0128 (16)	0.0048 (14)

### Geometric parameters (Å, º)

**Table:**

Cl1—C2	1.738 (2)	C4—C5	1.365 (3)
Cl2—C4	1.739 (3)	C5—C6	1.390 (3)
O1—C1	1.333 (3)	C5—H5	0.9300
O1—H1	0.9457	C6—C7	1.449 (3)
N1—C7	1.276 (3)	C7—H7	0.9300
N1—C8	1.468 (3)	C8—C9	1.530 (4)
C1—C2	1.397 (4)	C8—H8A	0.9700
C1—C6	1.414 (3)	C8—H8B	0.9700
C2—C3	1.366 (4)	C9—C9^i^	1.506 (5)
C3—C4	1.385 (3)	C9—H9A	0.9700
C3—H3	0.9300	C9—H9B	0.9700
			
C1—O1—H1	106.9	C5—C6—C7	120.1 (2)
C7—N1—C8	119.0 (2)	C1—C6—C7	119.9 (2)
O1—C1—C2	120.5 (2)	N1—C7—C6	121.8 (2)
O1—C1—C6	122.1 (2)	N1—C7—H7	119.1
C2—C1—C6	117.4 (2)	C6—C7—H7	119.1
C3—C2—C1	122.1 (2)	N1—C8—C9	111.1 (2)
C3—C2—Cl1	119.3 (2)	N1—C8—H8A	109.4
C1—C2—Cl1	118.6 (2)	C9—C8—H8A	109.4
C2—C3—C4	119.4 (3)	N1—C8—H8B	109.4
C2—C3—H3	120.3	C9—C8—H8B	109.4
C4—C3—H3	120.3	H8A—C8—H8B	108.0
C5—C4—C3	120.7 (2)	C9^i^—C9—C8	113.3 (3)
C5—C4—Cl2	120.5 (2)	C9^i^—C9—H9A	108.9
C3—C4—Cl2	118.9 (2)	C8—C9—H9A	108.9
C4—C5—C6	120.4 (2)	C9^i^—C9—H9B	108.9
C4—C5—H5	119.8	C8—C9—H9B	108.9
C6—C5—H5	119.8	H9A—C9—H9B	107.7
C5—C6—C1	120.0 (2)		
			
O1—C1—C2—C3	−178.1 (2)	C4—C5—C6—C7	178.9 (2)
C6—C1—C2—C3	1.5 (4)	O1—C1—C6—C5	178.1 (2)
O1—C1—C2—Cl1	0.4 (4)	C2—C1—C6—C5	−1.5 (4)
C6—C1—C2—Cl1	−180.0 (2)	O1—C1—C6—C7	−0.5 (4)
C1—C2—C3—C4	−0.4 (4)	C2—C1—C6—C7	179.9 (2)
Cl1—C2—C3—C4	−178.8 (2)	C8—N1—C7—C6	177.7 (2)
C2—C3—C4—C5	−0.9 (4)	C5—C6—C7—N1	−175.5 (2)
C2—C3—C4—Cl2	178.2 (2)	C1—C6—C7—N1	3.1 (4)
C3—C4—C5—C6	1.0 (4)	C7—N1—C8—C9	−93.8 (3)
Cl2—C4—C5—C6	−178.2 (2)	N1—C8—C9—C9^i^	−63.4 (4)
C4—C5—C6—C1	0.3 (4)		

Symmetry code: (i) −*x*, −*y*+1, −*z*+1.

### Hydrogen-bond geometry (Å, º)

**Table:**

*D*—H···*A*	*D*—H	H···*A*	*D*···*A*	*D*—H···*A*
O1—H1···N1	0.95	1.71	2.565 (3)	149
C5—H5···O1^ii^	0.93	2.48	3.370 (3)	160
C3—H3···Cl2^iii^	0.93	2.83	3.738 (3)	165

Symmetry codes: (ii) −*x*+1/2, *y*−1/2, *z*; (iii) −*x*+1, −*y*+1, −*z*.
